# Reducing Lung Biopsy Complications with Saline Injection: Evidence from a CT-Guided Cohort Study

**DOI:** 10.3390/diagnostics16091322

**Published:** 2026-04-28

**Authors:** Mohammed Khalaf, Yaroslava Von Rymon Lipinski, Sascha Herber

**Affiliations:** 1 Department of Neuroradiology, University Medical Center Mainz, Johannes Gutenberg University, 55131 Mainz, Germany; 2 Department of Radiology, Catholic Clinic Koblenz-Montabaur, 56073 Koblenz, Germany

**Keywords:** CT-guided intervention, needle trajectory, puncture angle, saline tract injection, pneumothorax, percutaneous procedure

## Abstract

**Purpose:** In this study, our aim is to assess the efficacy of saline-assisted needle withdrawal in minimizing pneumothorax and bleeding complications during CT-guided lung biopsies. **Methods:** In this retrospective study, 400 patients who underwent CT-guided lung biopsy were divided into two groups: 200 patients underwent conventional needle withdrawal (control group), and the other 200 patients underwent saline-assisted needle withdrawal (NaCl group). Needle angle, patient positioning, demographic data, and other procedural variables were collected. The primary outcome was the incidence of pneumothorax; secondary outcomes included bleeding rates and the impact of procedural factors on complication risk. Statistical analyses included Chi-square tests, logistic regression, and multinomial modeling. **Results:** The NaCl group demonstrated a significantly lower incidence of clinically significant pneumothorax (13.5%) compared to the control group (22.0%) (*p* = 0.007), while bleeding complications occurred in 26.5% of patients in the former versus 45.0% in the latter (*p* < 0.001). Multivariate analysis suggested a non-significant trend toward a higher pneumothorax risk with shallow needle angles (<60°; *p* = 0.502). Saline injection was especially advantageous in patients with underlying lung disease, reducing pneumothorax severity even when overall incidence rates were similar. No adverse events were attributed to the use of saline. **Conclusions:** Saline-assisted tract sealing is a safe and cost-effective technique that significantly reduces the risk of clinically significant pneumothorax and bleeding in CT-guided lung biopsies. Due to its simplicity and favorable safety profile, this approach holds considerable promise for widespread clinical implementation.

## 1. Introduction

Lung cancer is the most common cancer in males and the second most diagnosed cancer worldwide, with 2,480,301 new cases reported in 2022 [1]. It remains the leading cause of cancer mortality, responsible for 19% of all cancer deaths, totaling 1,817,172 fatalities in 2022 [1]. The implementation of advanced lung cancer screening programs is expected to increase the detection of early-stage lung cancer [2,3,4]. According to the National Comprehensive Cancer Network (NCCN) guidelines for Non-Small Cell Lung Cancer (NSCLC), any suspicious lung nodule should undergo pathological sampling before surgical intervention or stereotactic radiation therapy, provided the patient’s clinical status and nodule characteristics allow for the procedure to be undertaken [5]. In cases of advanced disease where surgical samples are unlikely to be available, obtaining adequate biopsy material is critical for accurate molecular profiling, particularly given the growing availability of targeted therapies [3,6,7]. The role of CT-guided lung biopsy in characterizing pulmonary nodules and ensuring appropriate diagnostic and therapeutic decisions is well-established.

Despite its value, CT-guided lung biopsy carries significant risks, with pneumothorax and bleeding being the most common. Recent meta-analyses report an overall complication rate of nearly 40% for CT-guided core lung biopsies, with pneumothorax occurring in over 25% of cases and 6% of these requiring chest tube insertion [8]. Results from the literature indicate that factors such as needle angle, patient positioning during the procedure, and patient demographics (age, sex) are known to influence both the procedural success and the likelihood of complications.

In an effort to mitigate the aforementioned risks, a variety of preventive strategies have been developed, encompassing techniques such as rapid roll-over, deep expiration and breath-holding, and the utilization of sealant materials such as autologous blood, collagen, hemocoagulase, or hydrogel plugs. These techniques have been demonstrated to reduce pneumothorax and the necessity for chest tube placement [9,10,11,12,13,14,15]. A less frequently explored method is saline injection during needle withdrawal, which entails the injection of a saline solution (NaCl 0.9%) through the coaxial needle concurrently with its withdrawal, thereby theoretically sealing the needle tract and minimizing air leakage into the pleural space. This method is cost-effective, readily available, and free from adverse reactions; however, evidence supporting its efficacy is limited [16,17,18,19,20,21]. In this study, our primary aim was to evaluate the efficacy of saline sealing in reducing complication rates, particularly pneumothorax and bleeding, during CT-guided lung biopsies. Secondary aims included assessing the impact on complication rates of procedural factors, such as needle angle and patient positioning, as well as patient-related variables, including age, sex, comorbidities, and platelet aggregation inhibition. We hypothesized that saline sealing would significantly reduce overall complications and that outcomes would vary according to procedural and demographic factors.

## 2. Methods

The Ethics Committee of the State Medical Association approved this retrospective study (approval number 2025-18430-retrospektiv, date: 12 October 2025). As this study was conducted retrospectively using anonymized clinical data, the requirement for individual patient informed consent was waived by the ethics committee in accordance with applicable institutional and national regulations. No additional interventions were performed on patients for research purposes.

This retrospective observational study included patients who underwent CT-guided lung biopsy.

### 2.1. Interventions

Patients were classified into two groups based on the chronological period of treatment. The control group comprised patients who underwent CT-guided lung biopsy with conventional needle withdrawal before the institutional adoption of the saline-assisted technique. The NaCl group comprised patients treated after the implementation of saline-assisted needle withdrawal as part of routine clinical practice. This temporal cohort design ensured that no patients were prospectively randomized or allocated to different techniques for research purposes; both techniques were applied as the standard of care at the respective time periods.

In the saline-assisted technique, approximately 10–20 mL of sterile normal saline (NaCl 0.9%) was injected through the coaxial needle during its withdrawal from the lung parenchyma. The injection was performed at a slow, continuous rate concurrent with needle withdrawal, and was terminated upon complete withdrawal of the coaxial needle from the chest wall. All operators followed a standardized departmental protocol to ensure uniformity of the technique across procedures.

### 2.2. Procedural Parameters

Procedural parameters included documentation of the needle angle in degrees and patient positioning during the procedure (supine, prone, or lateral).

### 2.3. Study Design

**Figure 1:**
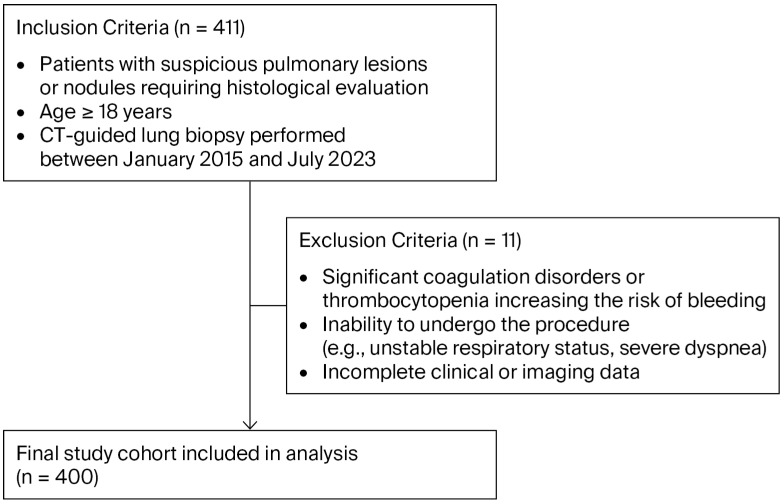
Study population inclusion and exclusion process.

This retrospective study was conducted at Catholic Clinic Koblenz-Montabaur between January 2015 and July 2023 using anonymized data extracted from the radiology information system from all patients who underwent CT-guided lung biopsy. The control group comprised consecutive patients biopsied during the period before the institutional adoption of the saline-assisted technique (January 2015 to December 2018), while the NaCl group comprised consecutive patients biopsied after the technique was introduced into routine clinical practice (January 2020 to July 2023). No patients were prospectively randomized; allocation to the two groups was determined solely by the time period of treatment. Figure 1 illustrates the patient inclusion and exclusion process.

#### 2.3.1. Study Population

A total of 400 patients who underwent CT-guided lung biopsy were included, with eligible patients including those with suspected pulmonary nodules requiring a biopsy. Patients were excluded if they had non-correctable coagulation disorders, such as an INR greater than 1.5 and a platelet count below 50 × 10^9^/L, or if they were unable to temporarily discontinue anticoagulant or antiplatelet therapy. Demographic data, including age and sex, as well as relevant clinical characteristics, were collected for all participants. Additionally, the distance of the target lesion from the pleural surface was recorded, distinguishing between pleural-based lesions and those with intervening normal lung parenchyma, as this factor may significantly influence complication risk. Lesion size and location were also documented.

#### 2.3.2. Outcome Measures

The primary outcome was complication rate, focusing on pneumothorax and bleeding. Pneumothorax was classified as clinically inapparent, defined as radiologically detected pneumothorax without associated clinical symptoms and not requiring therapeutic intervention, or clinically significant, defined as pneumothorax associated with clinical symptoms or requiring medical or interventional treatment, including chest tube placement. Bleeding was classified as radiographic bleeding, defined as peri-lesional or peri-procedural hemorrhage detected on post-biopsy CT without associated clinical symptoms, or clinically significant bleeding, defined as events accompanied by hemoptysis, a measurable decrease in hemoglobin, the need for blood transfusion, or the need for additional therapeutic intervention. Through secondary analyses, we assessed the impact of procedural variables, patient demographics, underlying lung disease, and platelet aggregation inhibition on complication rates.

### 2.4. Statistical Analysis

Statistical analyses were performed using IBM SPSS Statistics (version 29.0.2.0). Descriptive statistics summarized demographic and clinical characteristics, with continuous variables presented as mean ± standard deviation (SD) and range, and categorical variables as frequencies and percentages. Group differences in continuous variables were assessed using independent-samples *t*-tests, while categorical variables (e.g., gender, pneumothorax, and bleeding) were analyzed via Chi-square tests. A *p*-value < 0.05 was considered statistically significant.

To examine associations between pneumothorax categories (none, clinically inapparent, clinically significant) and predictors—needle angle, group allocation, age, gender, and patient position—a multinomial logistic regression was conducted, reporting odds ratios (ORs) with 95% confidence intervals (CIs). Underrepresented categories (e.g., needle angles > 120°) were excluded for model stability. To assess bleeding at the puncture site, binary logistic regression was performed with the same predictors. Results were reported as ORs with 95% CIs, with *p* < 0.05 indicating significance. This approach ensured robust identification of significant predictors for pneumothorax and bleeding.

## 3. Results

A total of 400 patients were included in this study: 200 in the control group, who did not receive a NaCl injection, and 200 in the NaCl-treated group.

Table 1 shows the age and gender distribution of both groups; there was no difference in gender distribution, but the NACL group was older (Table 1).

A comparison of the two groups revealed a significant difference in age but no significant difference in gender distribution (Table 1). The mean age in the control group was 67.6 ± 11.4 years, ranging from 32 to 90 years, while in the NaCl group it was 70.4 ± 10.1 years, ranging from 38 to 100 years. This age difference was statistically significant (*p* = 0.005), indicating that the NaCl group was older on average. In terms of gender distribution, 59.5% of the control group were men (119 individuals), while 40.5% were women (81 individuals); 53.5% were men (107 individuals) and 46.5% were women (93 individuals) in the NaCl group. This difference in gender distribution was not statistically significant (*p* = 0.23), suggesting that the groups were comparable in terms of gender composition.

The occurrence and severity of pneumothorax were then compared between the control and NaCl group, incorporating the updated dataset with additional cases of clinically significant pneumothorax in the latter. In the control group, 65.0% of patients (130 out of 200) exhibited no pneumothorax, 13.0% (26 out of 200) demonstrated a clinically inapparent pneumothorax, and 22.0% (44 out of 200) displayed a clinically significant pneumothorax. Conversely, in the NaCl group, the equivalent values were 63.5% (127 out of 200) of patients who hadno pneumothorax, 23.0% (46 out of 200), and 13.5% (27 out of 200), respectively. The incidence of pneumothoraces was comparable in both groups: 35% in the control and 36.5% in the NaCl group. Applying the Chi-square test to the statistical analysis revealed a significant difference in the distribution of pneumothorax categories between the control and NaCl groups (*p* = 0.007): the proportion of patients exhibiting clinically significant pneumothorax was found to be considerably lower in the latter when compared to the former (13.5% vs. 22.0%), thereby indicating a protective effect attributed to the administration of NaCl.

The mean distance is approximately 59.2 mm (5.92 cm), indicating the average depth across all measurements. The median is 60 mm (6.0 cm). The standard deviation is about 20 mm (2.0 cm), indicating a moderate spread of values with some variability in the dataset.

For the multinomial logistic regression analysis, cases with a value > 120° in the variable ‘angle of needle’ were excluded due to their low frequency (16 cases, 4.0% of the sample), as the limited sample size of this category had the potential to induce instability in the model, thereby compromising the reliability of the regression results. Consequently, the regression analysis was conducted using only cases with values “120–60°” and “<60°” for needle angle, representing 63.0% and 33.0% of the total sample, respectively (Table 2).

Through our regression analysis, we found no statistically significant difference in the risk of pneumothorax between needle insertion angles less than 60° and those between 60° and 120°. The odds ratios (OR = 0.95, 95%) suggested a trend toward a higher risk of pneumothorax with needle insertion angles less than 60°, although these differences were not statistically significant (*p*-value 0.502).

The following conditions were considered as underlying lung disease risk factors in our study: chronic obstructive pulmonary disease (COPD) and emphysema. These diagnoses were ascertained from clinical records, including discharge summaries and referring physician documentation, as well as CT imaging reports documenting emphysematous changes. Bleeding risk was assessed based on documented bleeding disorders, laboratory parameters (platelet count, INR), and relevant clinical history.

**Figure 2:**
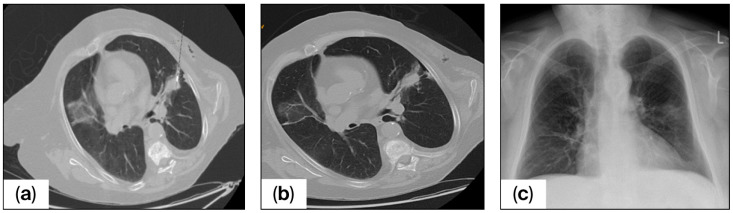
Axial non-contrast chest CT images in the lung window of a patient with a suspected pulmonary nodule in the left upper lobe. (**a**) The needle trajectory with an insertion angle of 48°. (**b**) Needle withdrawal after the biopsy tract was filled with saline solution, also revealing a small pneumothorax. (**c**) Chest X-ray performed two hours later showing no pneumothorax, and the patient remained asymptomatic throughout.

In this study, one of our key goals was mitigating pneumothorax risk in patients with diagnosed lung disease, including COPD and emphysema, as this has been identified as a significant concern and a primary area of focus for further investigation. In the control group, 93 patients were identified as having at least one risk factor, 23 out of 93 patients (24.7%) developed a pneumothorax as a complication following CT-guided puncture, and 19 cases (82.6%) were clinically significant pneumothoraces, while in the NaCl group, 85 patients had at least one risk factor, 26 out of 85 (30.5%, *p* = 0.4) developed pneumothorax after puncture, and 13 cases (50%) were classified as clinically significant. The analysis yielded a statistically significant difference between the two groups (*p* = 0.007). Figure 2 shows the target lesion and the puncture needle in situ on the planning CT and depicts the subsequent radiographic control following the procedure.

Furthermore, a comparison was made between the control and the NaCl group with regard to bleeding occurrence at the puncture site. In accordance with hospital protocol, platelet inhibition was to be continued with 100 mg acetylsalicylic acid and/or 75 mg clopidogrel, and the administration of medication containing oral anticoagulants or coumarins was suspended temporarily.

In the control group, 37% of patients (74 out of 200) exhibited platelet aggregation inhibition (*p* = 0.25) versus 31.5% (62 out of 200) in the NaCl group, revealing a statistically significant difference in the frequency of this complication between the two groups (*p* < 0.001). In the control group, bleeding occurred in 45.0% of cases (90 out of 200), while it was observed in only 26.5% (53 out of 200) in the NaCl group. Conversely, no bleeding was reported in 55.0% of the control group (110 out of 200) and in 73.5% of the NaCl group (147 out of 200).

The multinomial logistic regression analysis evaluated the influence of age, gender, and patient position on the occurrence of pneumothorax (see Table 3), revealing a borderline-significant association between age and clinically inapparent pneumothorax (odds ratio [OR] = 1.03, 95% confidence interval [CI] = 1.00–1.06, *p* = 0.052); however, no significant association was observed between age and clinically significant pneumothorax (OR = 1.01, 95% CI = 0.98–1.05, *p* = 0.078). The impact of gender and patient position on the likelihood of pneumothorax was investigated, finding that neither factor had a significant effect in any category (all *p* > 0.05).

In this study, we found that patients in the NaCl group exhibited a significantly lower probability of developing clinically significant pneumothorax when compared to the control group (OR = 0.378, 95% CI = 0.186–0.768, *p* = 0.007), thereby demonstrating a 71% reduction in risk.

The protective effect of NaCl administration was observed across all categories of pneumothorax. Specifically, the odds of no pneumothorax in the NaCl group were reduced by 39% in comparison with the control group (OR = 0.61, 95% CI = 0.45–0.81, *p* = 0.003), while those of clinically inapparent pneumothorax were reduced by 57% (OR = 0.43, 95% CI = 0.28–0.68, *p* < 0.001).

Concerning needle angles, a non-significant trend was observed suggesting that angles smaller than 60° may be associated with an increased likelihood of clinically significant pneumothorax compared to angles ranging from 60° to 120° (OR = 0.779, *p* = 0.502); however, this association did not reach statistical significance.

Patients with a history of at least one pre-existing lung disease exhibited an elevated risk of pneumothorax in both groups; however, the number of clinically relevant pneumothoraces was significantly lower in the NaCl group (*p* = 0.009).

Patients in the NaCl group exhibited a significantly lower probability of developing bleeding compared to the control group (OR = 0.404, 95% CI = 0.260–0.628, *p* < 0.001), thereby demonstrating a protective effect of NaCl administration. Furthermore, compared to males, female patients demonstrated a reduced likelihood of experiencing bleeding in comparison ([OR] = 0.594, 95% confidence interval [CI] = 0.384–0.920, *p* = 0.020).

Through this analysis we revealed that neither age (OR = 0.995, 95% CI = 0.976–1.015, *p* = 0.623) nor patient position (prone, supine, or lateral; all *p* > 0.05) had a significant influence on the probability of bleeding at the puncture site.

## 4. Discussion

The findings of this study demonstrate that saline-assisted needle withdrawal during CT-guided lung biopsies significantly reduces the incidence of clinically significant pneumothorax in comparison with conventional techniques. This reduction is clinically significant, as pneumothorax remains one of the most significant contributors to morbidity in patients undergoing percutaneous transthoracic needle biopsy (PTNB) [22,23,24]. While mild pneumothoraces are often self-limiting, moderate to severe cases typically necessitate chest tube insertion, which increases procedural burden, extends hospital stays, and adds to patient discomfort. Severe pneumothorax, albeit a rare complication, can progress to tension pneumothorax, a life-threatening condition caused by compression of the inferior vena cava, potentially resulting in sudden cardiovascular collapse [25].

Considering these risks, it is imperative to mitigate both the frequency and severity of pneumothorax to optimize patient outcomes. The necessity for efficacious strategies to mitigate the risk of pneumothorax is accentuated by the discrepancy between recommended thresholds and real-world data: The Society of Interventional Radiology (SIR) Quality Improvement Guidelines propose a threshold of 1% for pneumothorax necessitating chest tube insertion [26]; however, higher rates ranging from 5% to 6% [8] are consistently reported in the literature, emphasizing the necessity for innovative and accessible preventive techniques. A plethora of sealants have been explored in previous studies with varying success rates, including autologous blood, hydrogel, fibrin, and collagen plugs [9,10,11,12,13,14,15]. For instance, the BioSentry™ system, a self-expanding hydrogel plug, has demonstrated significant potential in reducing both pneumothorax rates and the necessity for chest tube placement in retrospective studies [27]. Nevertheless, such techniques are frequently associated with limitations, encompassing expenses, local tissue reactions, and the possibility of inflammation, which may constrain their extensive utilization.

Conversely, the NaCl sealing technique is a cost-effective and straightforward alternative. Normal saline is inexpensive, readily available, and does not elicit foreign body reactions, in contrast to other materials such as gelfoam, glue, or embolization coils. Applying this technology does not necessitate the use of additional devices or specialized training, thus rendering it well-suited for integration into standard clinical practice. Furthermore, the utilization of saline in our study exhibited a favorable safety profile, with no adverse reactions observed among patients [18].

The findings of the present study also demonstrate a significant reduction in bleeding at the puncture site among patients receiving saline injections, an effect likely attributable to the hydrostatic pressure exerted by saline during needle withdrawal, which may help tamponade vascular puncture sites even in patients with platelet aggregation inhibition. This outcome is of particular benefit to patients suffering from lung diseases or receiving anticoagulant therapy, where the risk of pneumothorax and bleeding is inherently higher [28]. However, the variation in pneumothorax presentation, with an increase in clinically inapparent pneumothoraces and a decrease in clinically significant cases, warrants further investigation. Whilst a clinically inapparent pneumothorax is less severe and frequently asymptomatic, it nonetheless requires monitoring, which has the potential to delay discharge.

It is imperative to comprehend the clinical significance of this transition in order to thoroughly assess the efficacy of NaCl sealing.

An important consideration when interpreting these results is the significant age difference between the two cohorts (70.4 ± 10.1 vs. 67.6 ± 11.4 years; *p* = 0.005). Older patients may be expected to have a higher baseline risk of complications due to age-related factors such as reduced lung elasticity and an increased prevalence of comorbidities including COPD and emphysema. The fact that the NaCl group, despite being significantly older, demonstrated fewer clinically significant pneumothoraces may in fact strengthen the argument for a genuine protective effect of the saline technique. Nevertheless, the borderline association between age and clinically inapparent pneumothorax (OR = 1.03, *p* = 0.052) warrants careful consideration, and future prospective studies should aim to match cohorts by age or employ stratified analyses to disentangle the effects of age from those of the intervention.

The observed shift from clinically significant to clinically inapparent pneumothoraces in the NaCl group likely carries genuine clinical significance, as it would be expected to translate into fewer chest tube placements, reduced or avoided hospitalizations, and decreased healthcare costs. However, a limitation of the present study is the absence of systematic data on chest tube placement rates, length of hospital stay, and the need for additional therapeutic interventions in both groups. Including such outcome data would substantially strengthen the clinical argument for saline tract sealing, and we strongly recommend that future prospective studies incorporate these clinically meaningful endpoints.

Regarding needle angle, our analysis revealed a non-significant trend toward a higher risk of pneumothorax with needle insertion angles less than 60° compared to those between 60° and 120° (OR = 0.779, *p* = 0.502). While this observation suggests a possible relationship between shallow needle trajectory and pleural injury, the association did not reach statistical significance and should therefore be interpreted with caution. The exclusion of cases with angles >120° (n = 16) due to low frequency further limits the generalizability of these angle-related findings. Future studies with larger sample sizes are needed to definitively evaluate the role of needle angle in complication risk [29]. Additionally, operator experience may play a role in the efficacy of NaCl sealing and complication rates. Given the temporal cohort design, improvements in overall procedural skill over time (learning curve effect) cannot be fully excluded as a confounding factor, highlighting the need for standardized training, protocols, and the incorporation of operator experience as an independent variable in future prospective studies [30].

Previous studies have also demonstrated the efficacy of saline injection in reducing pneumothorax rates [16,20,21]; however, the magnitude of reduction in both pneumothorax and bleeding rates observed in our study highlights the advantages of NaCl sealing, as its combination with other procedural strategies, such as rapid roll-over or forced expiration, has the potential to further enhance its effectiveness [18,21]. The strengths of our study include its large sample size, comprehensive evaluation of procedural and patient-specific factors (including needle angle, underlying lung disease, platelet aggregation inhibition, patient positioning, age, sex, and distance of the lesion from the pleural surface), and the use of multivariate regression to adjust for potential confounders. These considerations provide a nuanced understanding of the interplay between technique and outcomes.

The findings of this study should be interpreted within the context of several limitations. First, the retrospective design inherently carries the risk of selection and information bias, as incomplete or inconsistent records may have influenced data quality. Second, the two cohorts were assembled based on a chronological time period (before and after the adoption of the saline technique), rather than through randomization. This temporal design introduces the possibility that improvements in overall procedural skill, operator learning curves, or changes in clinical practice over time may have contributed to the observed differences, limiting causal inference regarding the effect of saline-assisted needle withdrawal. Third, the significant age difference between groups (*p* = 0.005) represents a potential confounding factor, although multivariate regression was employed to adjust for this variable. Fourth, a systematic assessment of individual operator experience was not performed, and variability in operator skill may have influenced complication rates. Fifth, systematic data on chest tube placement rates, length of hospital stay, and the need for additional therapeutic interventions were not available in our retrospective dataset, which limits the assessment of downstream clinical impact. Sixth, the lack of systematic long-term follow-up imaging data precludes evaluation of delayed complications.

The exclusion of cases with extreme needle angles (>120°) limits the generalizability of the findings. Additionally, the measurement of the needle angle is subject to a high degree of measurement inaccuracy and dependent variability. Finally, the subjective classification of pneumothorax severity highlights the need for standardized definitions to enhance comparability across studies.

The findings of this study suggest that NaCl sealing might prove useful in reducing complications during CT-guided lung biopsies. Its simplicity and accessibility render it especially appealing for settings with limited resources, where advanced sealant materials or imaging techniques may not be available. Implementing NaCl sealing within interventional radiology training curricula has the potential to standardize its utilization and enhance outcomes across various institutions.

Further studies are needed to validate our findings. Priority should be given to evaluating alternative pneumothorax prevention methods, particularly hydrogel plugs and autologous blood patches. Direct comparisons between saline sealing and these techniques are essential to determine the most effective approach. Additionally, exploring advanced imaging technologies, such as real-time or cone beam CT, may enhance procedural accuracy and reduce operator variability. Further research is also warranted to clarify the clinical significance of pulmonary hemorrhages, especially regarding the risks of pneumonia and significant periprocedural aspiration.

## 5. Conclusions

Our study demonstrates that NaCl tract sealing significantly reduces the incidence of clinically significant pneumothorax (OR 0.378, 95% CI 0.186–0.768, *p* = 0.007) and bleeding during CT-guided lung biopsies compared to a control group without NaCl. The technique is safe, simple, and cost-effective, making it suitable for widespread clinical implementation. Our results provide a solid foundation for future research aimed at optimizing biopsy procedures and improving patient outcomes.

## Figures and Tables

**Table 1:** Characteristics of both patient groups.

Variable	Control Group (*n* = 200)	NaCl Group (*n* = 200)	*p*
Age (years)			
Mean ± SD	67.6 ± 11.4	70.4 ± 10.1	0.005
Range	32–90	38–100	
Gender			
Men	119 (59.5%)	107 (53.5%)	0.23
Women	81 (40.5%)	93 (46.5%)	

**Table 2:** Distribution of needle angle categories.

Angle	Frequency
60–120°	252 (63%)
>120°	16 (4%)
<60°	132 (33%)
Total	400 (100%)

**Table 3:** Multinomial logistic regression results for bleeding. OR = odds ratio and 95% CI = 95% confidence interval.

Variable	Outcome	OR	95% CI	*p*-Value	Interpretation
Group (NaCl vs. Control)	Pneumothorax present	0.378	0.186–0.768	0.007	NaCl reduces the odds of pneumothorax by 62% compared to control; statistically significant
Age	Pneumothorax present	1.03	0.997–1.063	0.073	Slight increase in odds per year of age; not significant
Gender (Male vs. Female)	Pneumothorax present	0.925	0.463–1.847	0.825	No significant difference between males and females
Patient Position (Abdomen vs. Side)	Pneumothorax present	1.029	0.378–2.798	0.955	No significant effect
Patient Position (Back vs. Side)	Pneumothorax present	0.821	0.286–2.362	0.715	No significant effect
Needle Angle	Pneumothorax present	0.779	0.375–1.617	0.502	No significant effect

## Data Availability

The data supporting the findings of this study are available from the corresponding author upon reasonable request.
